# Bone turnover and periprosthetic bone loss after cementless total hip arthroplasty can be restored by zoledronic acid: a prospective, randomized, open-label, controlled trial

**DOI:** 10.1186/s12891-017-1577-2

**Published:** 2017-05-22

**Authors:** Tsan-Wen Huang, Chao-Jan Wang, Hsin-Nung Shih, Yuhan Chang, Kuo-Chin Huang, Kuo-Ti Peng, Mel S. Lee

**Affiliations:** 1 Department of Orthopaedic Surgery, Chang Gung Memorial Hospital, Chiayi, Taiwan; 2 Chang Gung University, Taoyuan, Taiwan; 3 Department of Orthopaedic Surgery, Chang Gung Memorial Hospital, Linkou, Taiwan; 4 Department of Diagnostic and Interventional Radiology, Chang Gung Memorial Hospital, Linkou, Taiwan; 5 Department of Orthopaedic Surgery, Kaohsiung Chang Gung Memorial Hospital, No. 123, Dapi Rd., Niaosong District, Kaohsiung City, 83301 Taiwan; 6 5 Fu-Hsin Street, Kweishan, Taoyuan, Taiwan; 7 No. 6, West Section, Chia-Pu Road, Pu-Tzi City, Chia-Yi Hsien 613 Taiwan; 8 No. 123, Dapi Rd., Niaosong District, Kaohsiung City, 83301 Taiwan

**Keywords:** Cementless total hip arthroplasty, Bone mineral density, Bisphosphonate, Stress shielding, Zoledronic acid, Bone turnover markers

## Abstract

**Background:**

Although the loss of bone mineral density (BMD) after total hip arthroplasty (THA) is a known problem, it remains unresolved. This study prospectively examined the effect of zoledronic acid (ZA) on bone turnover and BMD after cementless THA.

**Methods:**

Between January 2010 and August 2011, 60 patients who underwent cementless THA were randomly assigned to receive either ZA infusion or placebo (0.9% normal saline only) postoperatively. ZA was administered at 2 day and 1 year postoperatively. Periprosthetic BMD in seven Gruen zones was assessed preoperatively and at given time points for 2 years. Serum markers of bone turnover, functional scales, and adverse events were recorded.

**Results:**

Each group contained 27 patients for the final analysis. The loss of BMD across all Gruen zones (significantly in zones 1 and 7) up to 2 years postoperatively was noted in the placebo group. BMD was significantly higher in the ZA group than in the placebo group in Gruen zones 1, 2, 6, and 7 at 1 year and in Gruen zones 1, 6, and 7 at 2 years (*p* < 0.05). Compared with baseline measures of BMD, the ZA group had increased BMD in zones 1, 2, 4, 5, 6, and 7 at 1 year and in zones 1, 4, 6, and 7 at 2 years (*p* < 0.05). Serum bone-specific alkaline phosphatase and N-telopeptide of procollagen I levels were significantly increased at 6 weeks in the placebo group and decreased after 3 months in the ZA group. A transient decrease in osteocalcin level was found at 6 months in the ZA group. Functional scales and adverse events were not different between the two groups.

**Conclusions:**

The loss of periprosthetic BMD, especially in the proximal femur (zones 1 and 7), after cementless THA could be effectively reverted using ZA. In addition, bone turnover markers were suppressed until 2 years postoperatively following ZA administration.

**Trial registration:**

Chang Gung Memorial Hospital Protocol Record 98-1150A3, Prevention of Periprosthetic Bone Loss After Total Hip Replacement by Annual Bisphosphonate Therapy, has been reviewed and will be made public on ClinicalTrials.gov. Trial registration number: NCT02838121. Registered on 19 July, 2016.

## Background

Cementless total hip arthroplasty (THA) has become popular in recent decades [1, 2], but its long-term stability may be limited by progressive bone loss around the prosthetic implant [3, 4]. Periprosthetic bone loss is associated with reduced bone mineral density (BMD) in the periprosthetic Gruen zones [5–7] and may increase the risk of migration, implant loosening, and periprosthetic fractures [8].

A reduction in BMD, especially in the calcar region, is a common sequela of THA [6, 9–11]. Thus, preserving bone mineral content is important. Bisphosphonates are anti-resorptive agents that promote bone mineralization and inhibit farnesyl pyrophosphate synthase [12]. Their protective effects after joint arthroplasty have been shown in recent meta-analysis studies [13–15] wherein the periprosthetic BMD continued to increase by 9.40% at 18–70 months after discontinuation of bisphosphonate therapy.

Zoledronic acid (ZA), a third-generation bisphosphonate, is several times more potent than the first- or second-generation bisphosphonates. It is well tolerated and can rapidly lower bone turnover rates in children and adults at high risk of fractures [16–19]. In the pivotal Health Outcomes and Reduced Incidence with ZA Once-Yearly Trial (HORIZON-PFT), a once-yearly infusion of 5-mg intravenous ZA for a 3-year period significantly reduced the risk of vertebral fractures by 70%, hip fractures by 41%, and non-vertebral fractures by 25% in post-menopausal women with osteoporosis [20]. ZA protects against osteoporotic fractures [21]. Recently, two studies demonstrated the efficacy of ZA in patients who underwent cementless THA [22, 23], but ZA was administered on different schedules, leaving questions about the optimal dose timing of ZA unanswered.

In the present study, we administered ZA during the early postoperative period after cementless THA, and as a booster dose at 1 year postoperatively. The effects of ZA on periprosthetic BMD and functional outcome measures were assessed prospectively. Additionally, we examined safety concerns surrounding the dosing of ZA.

## Methods

### Study design

This prospective, randomized, open-label clinical trial was registered in ClinicalTrials.gov (NCT02838121). The Institutional Review Board of the study institution approved the study protocol (Reference number 98-1150A3), which adhered to the Declaration of Helsinki. All of the study participants provided written informed consent.

Based on Arabmotlagh et al. [10], the assumption of mean BMD change was −8% in the placebo group and 6% in the ZA group, with a standard deviation (SD) of 15%. Power analysis indicated that 25 patients were required, per group, to achieve a power of 0.9 with a 5% significance level. To avoid drop off and loss to follow-up, we recruited 30 patients in each group, for a total study sample of 60 patients. The assumptions were proven to be adequate because a similar sample size was reported by another prospective randomized trial [22]. Eligible patients were randomly assigned to either the ZA or placebo groups by an envelope drawing.

On the day following cementless THA, and at 1-year post-THA, the ZA group received 5 mg ZA (Aclasta®; Novartis Pharmaceuticals Corporation) via intravenous infusion with 0.9% normal saline (500 mL). The control group received only an intravenous saline infusion. All patients received oral calcium (600 mg) and vitamin D3 supplements (800 IU) daily throughout the course of the study [24]. Follow-up for radiographic and functional assessments was conducted at 2, 6, and 12 weeks, 6 months, and 1 and 2 years postoperatively.

### Patients

Patients aged 35–85 years undergoing THA, who received dual-energy x-ray absorptiometry (DXA) scanning within the 3 months preceding surgery, were considered for enrollment. Exclusion criteria included use of bisphosphonates during the preceding 2 years; uncontrolled seizure disorders; invasive malignancy within the preceding 5 years; osteogenesis imperfect; multiple myeloma; Paget’s disease; iritis; uveitis; diabetic neuropathy/retinopathy; active primary hyperthyroidism; Aspartate aminotransferase (AST), alanine aminotransferase (ALT), or bone-specific alkaline phosphatase levels more than twice the normal limit; serum calcium level >11 mg/dL; hypocalcemia; renal insufficiency (creatinine clearance ≤35 mL/min); use of investigational drugs; and the use of hip protectors or implants on the contralateral hip joint.

The patients’ demographic data, body mass index, pre-operative diagnoses, and baseline characteristics were recorded.

### Total hip arthroplasty

All of the patients underwent standardized THA via direct lateral approach [25, 26] using a Zimmer Trilogy Cup, VerSys Fiber Metal Taper Stem, and highly cross-linked polyethylene, coupled with a 32 mm metal head. An experienced surgeon performed all of the procedures. Based on our standard of care following cementless THA, the patients were encouraged to ambulate as soon as possible after surgery and advised to protect against weight bearing for 6 weeks.

### Assessments

An experienced clinician, blinded to group assignment and patients’ demographic data, performed all radiographic and clinical assessments.

On each follow-up visit, radiographic evaluation of the total hip prosthesis was performed on each standard antero-posterior views of the pelvis and lateral views of the operated hip according to methods described by Engh et al. [27] and Johnston et al. [28]. The vertical distance between the lateral shoulder of the prosthesis and the superior tip of the greater trochanter on the radiograph was measured. This served as the reference distance for monitoring implant migration. At each study visit, this distance was measured and recorded.

The patients underwent DXA scanning of the operated hip using a densitometer (Hologic Inc., Waltham, MA) for quantifying bone mass and density changes [7]. To estimate the precision of the densitometer, double measurements involving repositioning of the patient and the scanner between the first and second scans were made in 10 patients [29]. The DXA method had a measurement error of 1%–3% in Gruen zones. BMD was measured in the frontal plane, throughout seven Gruen zones, and changes in BMD ratios from baseline were estimated for each zone.

Functional assessments included the Hip Harris Score (HHS), UCLA activity score, Short-Form (SF)-12 Physical Component Summary (PCS), and SF-12 Mental Component Summary (MCS). Renal function (glomerular filtration rate [GFR] and creatinine level), hepatic function (AST and ALT), serum calcium, and levels of bone turnover biomarkers (osteocalcin, bone-specific alkaline phosphatase, and N-telopeptide of procollagen I) were also assessed [30, 31].

Complications, including reported need for analgesics, were recorded. Any medical or surgical event that compromised clinical recovery was defined and recorded as an adverse event. A relatively poorer and slower functional recovery beyond 3 months and an HHS score <80 were considered adverse events.

The primary endpoint was the change in periprosthetic BMD, between baseline and all other time points. Secondary endpoints included radiologic analyses, implant migration, levels of serum markers for bone metabolism, functional outcomes, and safety and tolerability of the experimental drug.

### Statistical analysis

Values are presented as mean (±SD). Group differences were analyzed using independent-samples *t*-test. Time-based differences were analyzed by repeated measures analysis of variance (ANOVA) using a general linear model with Greenhouse-Geisser correction. Significance was set at *p* < 0.05. Post hoc comparisons were performed using Bonferroni corrections for multiple comparisons. SPSS software version 13.0 (SPSS Inc., Chicago, IL) was used for all analyses.

## Results

### Patient demographics

**Fig. 1:**
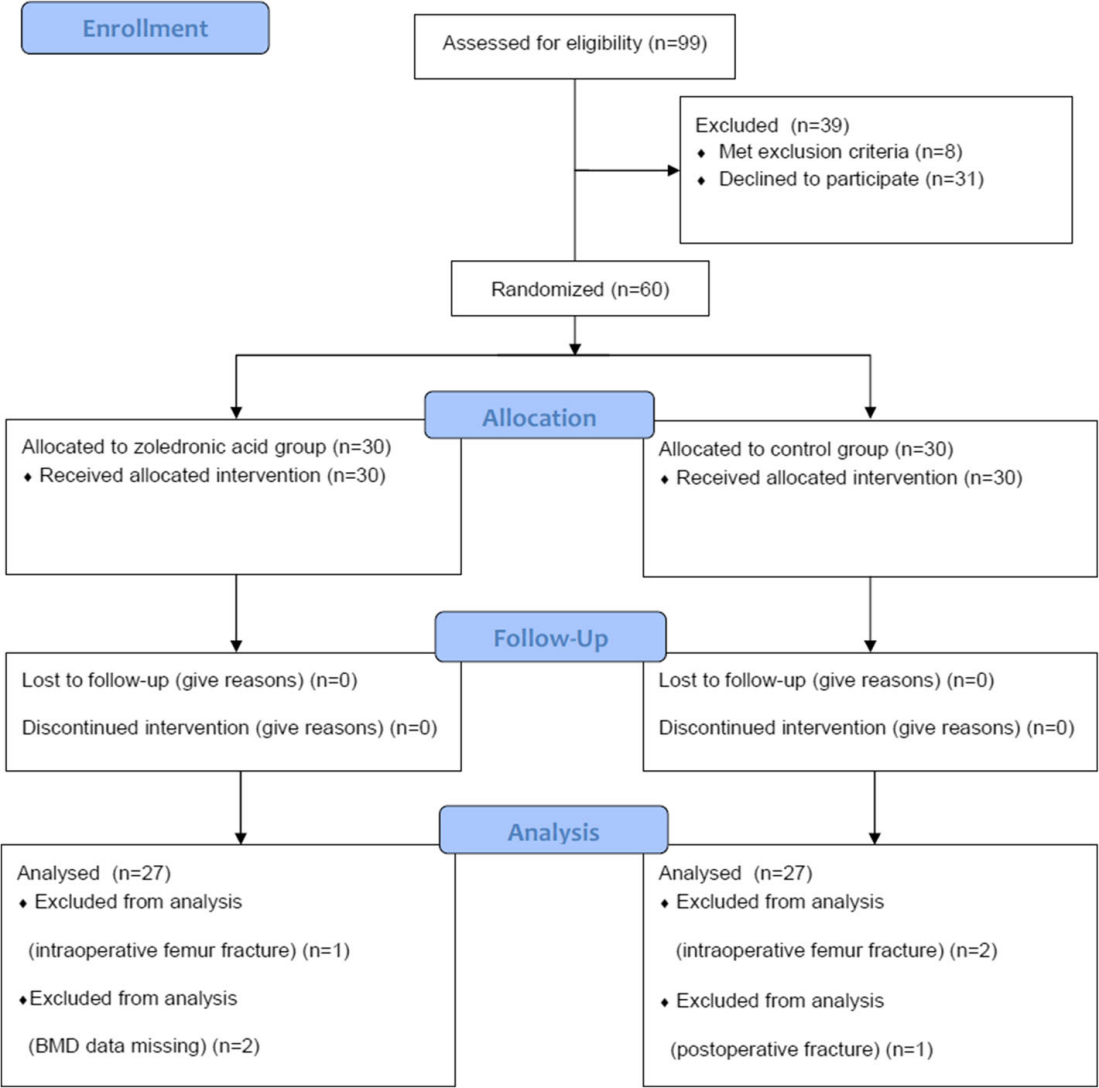
Patient disposition

Between January 2010 and August 2011, 60 patients were enrolled. Following randomization into either the ZA or placebo control groups, four patients (one in the ZA group and three in the control group) were excluded due to periprosthetic fractures and an additional two (both from the ZA group) were excluded due to missing BMD data at 1 year (Fig. 1). Each group had 27 patients for the final analysis and no significant differences in baseline characteristics were found between the two groups (Table 1).

**Table 1:** Patient demographics and surgical results

	ZA group (*n* = 27)	Control group (*n* = 27)
Age (years), mean (SD)	60.1 (11.7)	59.4 (13.3)
Sex, female:male	15:12	14:13
BMI (kg/m^2^), mean (SD)	26 (4)	25 (5)
Operation time (minutes), mean (SD)^a^	118 (27)	103 (17)
Blood loss (mL), mean (SD)	502 (312)	387 (162)
Diagnosis (OA/AVN)	16/11	17/10

*BMI* body mass index, *SD* standard deviation, *ZA* zoledronic acid, *OA* osteoarthritis, *AVN* avascular necrosis

^a^statistically significant difference between the ZA and control groups (*P* < 0.05)

### Radiographic analysis

All implants showed stable osteo-integration without evidence of early or late migration. There were no radiolucent lines at the prosthesis-bone interface of the cups and stems, and no pedestal formation in any stem, in either group.

### Bone mineral density

At baseline, both groups had similar BMD. The delta BMD at each time point revealed that at 12 weeks, the ZA group had significantly higher BMD than the control group in Gruen zones 2, 6, and 7. Increases in BMD persisted at 6 months in zones 6 and 7, at 1 year in zones 1, 2, 6, and 7, and at 2 years in zones 1, 6, and 7 (Table 2). The BMD changes from baseline (BMD ratio) were significantly higher for the ZA group in Gruen zones 2, 4, 6, and 7 at 12 weeks; in zones 1, 6, and 7 at 6 months; in zones 1, 2, 4, 5, 6 and 7 at 1 year, and in zones 1, 4, 6, and 7 at 2 years (Table 3).

**Table 2:** Mean (SD) BMD (g/cm^2^) for both groups in all gruen zones

Gruen zones	1	2	3	4	5	6	7	Delta
Baseline								
ZA group	0.64(0.14)	1.26(0.21)	1.52(0.24)	1.63(0.19)	1.57(0.19)	1.24(0.21)	0.96(0.19)	1.15(0.16)
Control	0.64(0.16)	1.24(0.22)	1.53(0.21)	1.66(0.21)	1.61(0.23)	1.25(0.22)	0.97(0.24)	1.15(0.17)
*p*-value	0.863	0.778	0.779	0.522	0.446	0.951	0.830	0.968
12 weeks								
ZA group	0.74(0.38)	1.46(0.28)	1.69(0.88)	1.73(0.35)	1.75(0.58)	1.40(0.21)	1.09(0.22)	1.38(0.60)
Control	0.59(0.14)	1.28(0.21)	1.50(0.21)	1.61(0.21)	1.61(0.22)	1.23(0.23)	0.88(0.20)	1.16(0.14)
*p*-value	0.060	0.010^a^	0.276	0.142	0.238	0.009^a^	0.001^a^	0.077
6 months								
ZA group	0.65(0.14)	1.40(0.17)	1.55(0.17)	1.68(0.19)	1.62(0.27)	1.40(0.19)	1.04(0.25)	1.26(0.12)
Control	0.59(0.15)	1.31(0.26)	1.51(0.22)	1.62(0.23)	1.63(0.23)	1.26(0.23)	0.84(0.30)	1.17(0.16)
*p*-value	0.109	0.144	0.529	0.301	0.922	0.021^a^	0.008^a^	0.021^a^
1 year								
ZA group	0.66(0.15)	1.38(0.19)	1.50(0.24)	1.68(0.19)	1.66(0.16)	1.40(0.22)	1.01(0.27)	1.25(0.13)
Control	0.54(0.20)	1.19(0.38)	1.44(0.38)	1.54(0.38)	1.51(0.39)	1.20(0.36)	0.80(0.30)	1.11(0.28)
*p*-value	0.021﻿^a^	0.035^a^	0.434	0.096	0.057	0.014^a^	0.011^a^	0.021^a^
2 years								
ZA group	0.67(0.15)	1.38(0.20)	1.49(0.25)	1.68(0.19)	1.65(0.20)	1.44(0.21)	1.01(0.27)	1.26(0.13)
Control	0.55(0.15)	1.35(0.77)	1.51(0.23)	1.59(0.23)	1.62(0.21)	1.21(0.24)	0.78(0.22)	1.14(0.15)
*p*-value	0.013^a^	0.870	0.786	0.167	0.612	0.001^a^	0.003^a^	0.011^a^

^a^statistically significant difference between ZA and control groups

*BMD* bone mineral density, *SD* standard deviation, *ZA* zoledronic acid

**Table 3:** Mean (SD) bone mineral density ratio (the BMD changes from baseline) for each group by gruen zone at different time points

Gruen zones	1	2	3	4	5	6	7
12 weeks							
ZA group	1.20(0.74)	1.18(0.28)^a^	1.13(0.62)	1.07(0.23)^a^	1.13(0.41)	1.13(0.13)^a^	1.15(0.21)^a^
Control	0.93(0.14)	1.04(0.12)	0.98(0.08)	0.97(0.04)	1.00(0.07)	1.00(0.15)	0.93(0.22)
6 months							
ZA group	1.05(0.19)^a^	1.13(0.12)	1.05(0.21)	1.04(0.07)	1.04(0.18)	1.14(0.12)^a^	1.09(0.20)^a^
Control	0.92(0.18)	1.07(0.19)	0.99(0.08)	0.97(0.06)	1.01(0.07)	1.02(0.18)	0.88(0.29)
1 year							
ZA group	1.05(0.19)^a^	1.10(0.10)^a^	1.00(0.09)	1.03(0.08)^a^	1.07(0.12)^a^	1.13(0.13)^a^	1.07(0.22)^a^
Control	0.86(0.24)	0.98(0.25)	0.95(0.22)	0.93(0.20)	0.95(0.24)	0.97(0.28)	0.84(0.29)
2 years							
ZA group	1.08(0.21)^a^	1.12(0.11)	1.00(0.11)	1.04(0.09)^a^	1.08(0.15)	1.16(0.13)^a^	1.06(0.22)^a^
Control	0.89(0.14)	1.10(0.46)	1.00(0.08)	0.96(0.07)	1.02(0.10)	1.00(0.19)	0.83(0.20)

^a^statistically significant difference between the ZA and control groups (*P* < 0.05)

*BMD* bone mineral density, *SD* standard deviation, *ZA* zoledronic acid

**Fig. 2:**
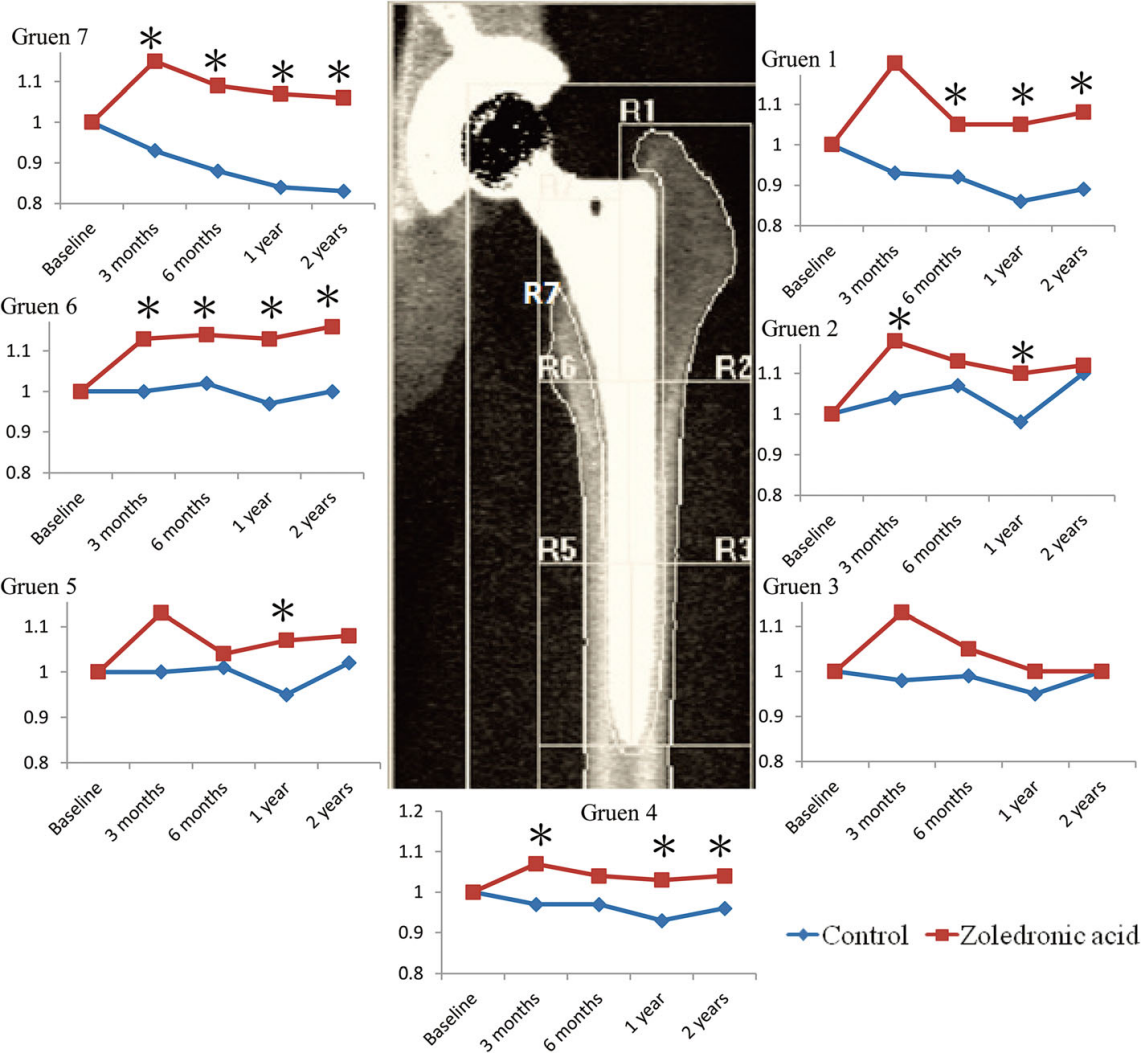
Bone mineral density changes in the zoledronic acid and control groups in Gruen zones

Time-based BMD differences in each Gruen zones were analyzed by repeated measure ANOVA (Fig. 2). In zone 1, the mean BMD change was 111% in the ZA group and 88% in the control group (95% CI, 10%–36%; *p* = 0.001). In zone 2, the mean BMD change was 114% (ZA) and 103% (control) (95% CI, 2%–20%; *p* = 0.018). In zone 4, the mean BMD change was 105% (ZA) and 95% (control) (95% CI, 5%–15%; *p* = 0.001). In zone 5, the mean BMD change was 108% (ZA) and 99% (control) (95% CI, 1%–18%; *p* = 0.024). In zone 6, the mean BMD change was 114% (ZA) and 98% (control) (95% CI, 7%–25%; *p* = 0.001). In zone 7, the mean BMD change was 110% (ZA) and 84% (control) (95% CI, 14%–37%; *p* < 0.001).

### Functional outcomes

There were no significant differences in HHS, SF-12 (PCS), SF-12 (MCS), and UCLA scores between the groups at any point in the study. However, the within-group functional scores changed significantly throughout the study period (*p* < 0.001) (Table 4). HHS increased significantly from baseline to 6 weeks, and thereafter up to 2 years, in both groups (*p* < 0.001). SF-12 (PCS) scores were lower at 2 weeks compared to baseline (*p* < 0.001), but increased significantly from baseline to 12 weeks, and thereafter up to 2 years, in both groups (*p* < 0.001). The SF-12 (MCS) scores of both groups were significantly lower at 1 year compared to 6 weeks (*p* < 0.05). Compared to baseline, the UCLA scores of both groups were significantly lower at 2 and 6 weeks (*p* < 0.01), but significantly increased by 1 year (*p* < 0.001).

**Table 4:** Clinical assessments in each group at different time intervals (*n* = 54)

	Preop		2 week		6 week		3 months		6 months		1 year		2 years	
	N	mean ± SD	N	mean ± SD	N	mean ± SD	N	mean ± SD	N	mean ± SD	N	mean ± SD	N	mean ± SD
Harris hip score														
Group ZA	27	60.31 ± 10.49	27	56.23 ± 14.06	27	70.96 ± 13.03^a^	27	77.93 ± 10.63^a^	27	83.60 ± 7.52^a^	27	86.34 ± 7.08^a^	27	90.18 ± 2.11^a^
Group N/S	27	60.88 ± 11.63	27	58.80 ± 10.13	27	71.08 ± 12.26^a^	27	79.89 ± 9.42^a^	27	81.50 ± 9.87^a^	27	85.16 ± 7.28^a^	27	87.67 ± 5.44^a^
SF-12(PCS)														
Group ZA	27	27.53 ± 10.13	27	18.96 ± 7.48^a^	27	28.23 ± 11.35	27	40.01 ± 13.54^a^	27	48.09 ± 6.95^a^	27	51.29 ± 4.45^a^	27	53.69 ± 3.79^a^
Group N/S	27	29.16 ± 11.31	27	21.37 ± 8.34^a^	27	27.79 ± 11.99	27	42.12 ± 10.61^a^	27	48.96 ± 7.90^a^	27	50.53 ± 6.95^a^	27	52.50 ± 4.16^a^
SF-12(MCS)														
Group ZA	27	57.65 ± 12.57	27	60.87 ± 9.51	27	59.23 ± 10.59	27	60.3 ± 6.86	27	58.85 ± 4.95	27	58.49 ± 4.07	27	58.61 ± 5.98
Group N/S	27	59.17 ± 11.99	27	60.08 ± 7.29	27	63.72 ± 3.84	27	59.9 ± 4.77	27	55.97 ± 8.34	27	56.96 ± 7.20	27	59.01 ± 4.05
UCLA activity score														
Group ZA	27	3.96 ± 1.16	27	2.56 ± 0.80^a^	27	3.41 ± 0.97^a^	27	4.22 ± 0.93	27	4.63 ± 1.15	27	5.74 ± 1.06^a^	27	7.00 ± 0.90^a^
Group N/S	27	4.33 ± 1.59	27	2.56 ± 0.70^a^	27	3.44 ± 0.97^a^	27	4.52 ± 0.94	27	4.70 ± 0.95	27	5.52 ± 1.19^a^	27	6.50 ± 0.74^a^

Statistically significant difference between ZA and control groups

^a^significant difference between each time point and baseline

*SD* Standard deviation, *ZA* zoledronic acid, *SF* short form, *PCS* physical component summary, *MCS* mental component summary, *N/S* normal saline

### Renal and hepatic function and serum calcium levels

There were no significant differences in creatinine levels between the two groups at baseline or at any time point throughout the study. GFR increased within both groups between baseline and 6 months (*p* < 0.01) and baseline and 1 year (*p* < 0.001) (Table 5). The AST and ALT values at baseline and at 6 and 12 weeks were within normal limits and had no group differences were realized at any time point. Serum calcium levels were similar in both groups at all time points.

**Table 5:** Mean serum creatinine (Cr) level, glomerular filtration rate (GFR), and bone turnover biomarkers in the ZA (zoledronic acid) and control groups as a function of time

	Baseline	6 weeks	12 weeks	6 months	1 year	2 years
Renal function						
*Cr (mg/dL)*						
ZA group	0.79 (0.23)	0.76 (0.27)	0.81 (0.28)	0.85 (0.59)	0.78 (0.24)	0.76 (0.26)
Control	0.83 (0.2)	0.81 (0.22)	0.79 (0.18)	0.79 (0.19)	0.76 (0.23)	0.79 (0.22)
*GFR (mL/min)*						
ZA group	59.39 (2.21)	60.08 (5)	61.84 (10.78)	77.56 (27.24)	83.8 (28.48)	93.9 (23.3)
Control	59.63 (1.28)	59.78 (1.15)	63.16 (12.83)	73.82 (23.6)	79.45 (25.96)	87.3 (19.5)
Ca (*mg/dL*)						
ZA group	9.53 (0.49)	9.34 (0.57)	9.52 (0.44)	9.46 (0.46)	9.43 (0.49)	9.37 (0.39)
Control	9.57 (0.43)	9.2 (1.77)	9.54 (0.36)	9.47 (0.52)	9.13 (1.89)	9.24 (0.29)
Bone turnover biomarkers						
*Bone-specific alkaline phosphatase (μg/L)*						
ZA group	78.6 (17.7)	77.3 (19.2)^a^	65.6 (11.7)^b^	67.5 (26.6)	68.7 (24.5)	66.5 (18.1)
Control	76 (17.6)	89.4 (21.7)	83.3 (22.8)	74.5 (19.9)	73.2 (22.9)	74 (15.9)
*Osteocalcin (ng/mL)*						
ZA group	21.1 (8.7)	18.1 (7.9)	17.2 (10.2)	14.7 (5)	16.8 (7.2)	14.9 (7.2)
Control	18.5 (10.3)	20.9 (13.2)	20.8 (11.5)	17.7 (6.4)	20.5 (10.4)	18.8 (8.6)
*N-telopeptide of procollagen I (ng/mL)*						
ZA group	53.9 (23.1)	57.1 (24.6)^a^	38.8 (16.9)^b^	30.5 (11.9)^b^	34.6 (19.5)	28.3 (11.3)^a^
Control	43.2 (21.8)	80.7 (44)	69.7 (38)	57.5 (24.9)	50.4 (35.4)	44.3 (19.3)

^a^significant difference between groups (*P* < 0.05)

^b^significant difference between groups (*P* < 0.001)

### Biomarkers of bone turnover

There was a significant reduction, from baseline, in levels of bone-specific alkaline phosphatase for the ZA group at 12 weeks, 6 months, 1 year, and 2 years postoperatively (*p* < 0.01). In the control group, bone-specific alkaline phosphatase was significantly increased at 6 and 12 weeks postoperatively (*p* < 0.05) (Table 5). In the ZA group, osteocalcin was significantly reduced between baseline and 6 months, 1 year, and 2 years (*p* < 0.05). The control group demonstrated significant increases in osteocalcin between baseline and 1 year (*p* < 0.05).

In the ZA group, levels of N-telopeptide of procollagen I were significantly reduced from baseline at 12 weeks, 6 months, 1 year, and 2 years postoperatively (*p* < 0.05). In the control group, these levels significantly increased from baseline at 6 weeks, 12 weeks, and at 6 months postoperatively (*p* < 0.05). There were significant differences in bone turnover biomarkers between the ZA and control groups (Table 5).

### Adverse events

Complications included fever (*n* = 3) and hypocalcemia (*n* = 1) but were mild-to-moderate in severity, and believed to be related to the investigational drug. No patient sustained osteonecrosis of the jaw or atypical femoral fracture. Three patients (one in the ZA group and two in the control group) had intra-operative periprosthetic fractures, and one (control) subject had postoperative periprosthetic fracture. These individuals were excluded from the final analysis.

## Discussion

The present study shows that ZA infusions on the day after cementless THA, and as a booster at 1 year postoperatively, significantly reduced periprosthetic femoral BMD loss. In the control group, BMD decreased significantly in Gruen zones 1 and 7 up to 2 years postoperatively. In contrast, for the ZA group, BMD significantly increased in all Gruen zones (except for zone 3) between baseline and 1 year postoperatively. At 2 years postoperatively it remained significantly increased in zones 1, 6, and 7. The greatest effect of ZA was observed in the proximal femur at 2 years, with BMD changes of +6% (control: −17%) in zone 7 and +8% (control: −11%) in zone 1. However, this improvement does not necessarily reflect better functional outcomes.

Only two reports have previously studied ZA in patients after cementless THA. Scott et al. [23] reported that ZA significantly prevented BMD loss in Gruen zones 1, 4, and 6 at 1 year, and in zones 1 and 6 at 2 years. However a decrease in BMD was still evident in zone 7 (calcar region) at 1 and 2 years. Our results were different. We found increased BMD after ZA across all Gruen zones at 1 year (though this difference did not rise to the level of statistical significance for zone 3), including Gruen zone 7. We thought the difference may be related to differences in Scott et al.’s timing of ZA administration (2 weeks postoperatively), or differences among the various types of prosthesis (two types of modular stems). The present study always administered ZA at 2 days postoperatively and used only one type of prosthesis. Periprosthetic BMD varies as a function of stem type. Patients with large and stiff femoral stems experience greater stress shielding, which results in more resorption of the proximal femur [15]. The modular femoral prosthesis has a larger proximal implant segment (for the neck-body taper junction) and is stiffer proximally. Proximal stiffness mismatches may increase proximal femoral stress shielding and subsequent bone loss. Moreover, more proximal bone loss occurs in femur preparation for larger femoral prosthesis. A femur with less bone mass is less stiff, relative to the implanted stem, and this will increase stress-related bone resorption [32]. In this study, we used a flat and double-tapered non-modular femoral stems. This design decreases the cross-sectional area moment of inertia and achieves initial stability by wedging into the proximal femur. It is considerably less stiff than the modular design and prevents periprosthetic bone loss.

In the second study, ZA (4 mg) was administered on the first postoperative day to 25 patients after cementless THA. Periprosthetic BMD was not analyzed but ZA improved the initial fixation of the cementless implant and prevented early implant migration, compared to 24 control patients [22]. The current study also used ZA in the acute postoperative period and did not find severe adverse events related to the drug. ZA infusion was deemed safe during the acute phase after THA. Taken together, early timing for postoperative ZA treatment may be a safe and effective means of preventing BMD loss after cementless THA.

Since periprosthetic bone loss is most pronounced in the early postoperative period [9], administering bisphosphonates soon after surgery is logical [22]. Ericksen and colleagues reported that the timing of the first ZA infusion changes BMD for patients who recently underwent hip surgery [33]. At 1 year postoperatively, all patients treated with ZA had increased BMD except for the group that received an early dose (≤2 weeks postoperatively). This group also demonstrated worse anti-fracture outcomes. While the current findings seem to contradict those of Ericksen et al., the patients in their study who received ZA ≤2 weeks after surgery had a higher risk of mortality due to older age and a greater number of co-morbidities. Moreover, their study had a smaller sample size and therefore group heterogeneity may have affected their findings. The current study provides a consolidated examination of BMD and revealed biochemical data supporting the use of ZA in the early postoperative period (2 days postoperatively). Since THA or bipolar hemiarthroplasty are common surgeries for patients with hip fractures, early initiation of ZA or other bisphosphonates is practical and may lead to superior outcomes.

In their meta-analysis, Bhandari et al. [13] suggested that bisphosphonates had a beneficial effect in maintaining periprosthetic BMD after THA or total knee arthroplasty. Arabmotlagh et al. [10] reported long-standing beneficial effects of alendronate in the prevention of periprosthetic bone loss 6 years after cementless THA. A single-dose of ZA can restore BMD beyond 1 year, with an effect comparable to that obtained with three annual ZA infusions [34]. In addition, ZA can prevent bone loss for long time periods. The HORIZON-PFT study reports a 6.02% increase in total hip BMD in patients, at risk of hip fractures, following 3 years of annual ZA treatment [19, 35]. Improvements in BMD continued for 6 years in these patients, suggesting a lasting effect of ZA.

Measuring serum calcium levels during ZA treatment is recommended [23]. In this study, both groups were administered oral calcium supplements. Serum calcium levels were within normal range throughout the study period (Table 5 **)**. Since ZA can lead to reduced serum calcium levels 6 weeks after its administration, providing calcium supplements is imperative.

Previous studies report short-term increases in creatinine after treatment, which typically resolve quickly, and without long-term impact on renal function [35]. In this study, there were no significant differences in renal function between the ZA and control groups, supportive of improved GFR over time.

This study found that bone turnover markers were significantly altered by the administration of ZA. Decreased bone turnover markers may contribute to the development of complications such as bisphosphonate-induced osteonecrosis of the jaw (ONJ) [23, 36] or atypical femoral fractures [37, 38]. ZA was administered over two doses in this study. This dosing was different from past studies of fracture prevention or studies that reported on complications such as ONJ and atypical femoral fractures related to prolonged use of bisphosphonates. Extending the current study period will be beneficial since our data revealed changes in bone turnover markers toward the conclusion of the study. By following patients for more than 2 years, we may learn more about these changes, as well as the risks of bisphosphonate-related complications.

The main limitation of this study was the sample size. Nonetheless, the findings are compelling and consistent with those of previous reports [12, 18, 20]. Although this study was powered to show changes in BMD throughout various Gruen zones, at various time points postoperatively, some zones exhibited borderline significances. It is possible that the study was underpowered and a larger sample size may be needed to demonstrate significant changes in BMD following administration of ZA. Bone mineral homeostasis correlates with the calcium and vitamin D metabolism. Another limitation of the current study was our failure to measure vitamin D levels at baseline. Although all study subjects had normal BMDs, labs, and no osteoporosis, we cannot rule out the possibility of an underlying vitamin D deficiency in study subjects. A future studies should clarify the effects of vitamin D levels.

## Conclusions

In conclusion, initiation of ZA treatment in the acute postoperative period preserves periprosthetic BMD in the proximal femur (zones 1 and 7). ZA may be effective prophylaxis against periprosthetic bone loss and implant migration. Future large-scale, longitudinal studies are necessary to demonstrate clinical effectiveness and investigate the risk of treatment-related complications.

## Abbreviations

ALT: Alanine aminotransferase
ANOVA: Analysis of variance
AST: Aspartate aminotransferase
BMD: Bone mineral density
DXA: Dual-energy x-ray absorptiometry
GFR: Glomerular filtration rate
HHS: Hip Harris Score
MCS: Mental Component Summary
PCS: Physical Component Summary
SD: Standard deviation
SF-12: Short-Form-12
THA: Total hip arthroplasty
ZA: Zoledronic acid
